# Mini review: A reevaluation of nutritional vitamin D in the treatment of chronic kidney disease

**DOI:** 10.1097/MD.0000000000035811

**Published:** 2023-10-27

**Authors:** Yingjing Shen

**Affiliations:** a Department of Nephrology, Shanghai Tianyou Hospital, Shanghai, China.

**Keywords:** chronic kidney disease-mineral and bone disorder, osteoporosis, therapy, vascular calcification, vitamin D deficiency

## Abstract

Chronic kidney disease-mineral and bone disorder is a syndrome of mineral and bone metabolism abnormalities caused by chronic kidney disease. Osteoporosis is a systemic metabolic bone disease characterized by low bone mass, disruption of bone microstructure, increased brittleness, and a higher propensity for fractures. Both of these conditions significantly affect bone metabolism and substantially increase the risk of fractures. Nutritional vitamin D is an essential trace element in the human body and an important fat-soluble vitamin. One crucial physiological role of nutritional vitamin D is to achieve mineral-bone metabolism balance by regulating calcium homeostasis. This review summarized the metabolism of vitamin in normal population and its specificity in chronic kidney disease. Over the years, the understanding and application of vitamin D in patients with chronic renal failure is changing. As people pay more attention to hypercalcemia, vascular calcification, osteoporosis, nutritional vitamin D has come into people’s attention again. More and more studies are discussing how to prescribe vitamin D supplementation in hemodialysis patients.

## 1. Introduction

Chronic kidney disease-mineral and bone disorder (CKD-MBD) is a syndrome of abnormal mineral and bone metabolism due to chronic kidney disease with one or more of the following clinical manifestations: abnormalities in calcium, phosphorus, parathyroid hormone (PTH), or vitamin D metabolism; abnormalities in bone turnover, mineralization, mass, linear growth, or strength; and calcification of blood vessels or other soft tissues.^[1]^ Osteoporosis (OP) is a systemic disorder of bone metabolism characterized by low bone mass and destruction of bone microarchitecture, which increases fragility and susceptibility to fracture.^[2]^ Both of these diseases severely affect bone metabolism and significantly increase the risk of fracture.

The prevalence of OP is higher in patients with CKD than in the general population,^[3,4]^and also varies depending on the stage of CKD, gradually increasing with decreasing renal function (*P* < .05).^[5,6]^ Fractures in patients with early CKD are more similar to those in conventional OP than in CKD-MBD.^[7]^ Most patients with CKD stages 4 to 5 have diminished mineral regulation by the kidney, therefore, often exhibit decreased bone mineral density (BMD) or varying degrees of CKD-MBD.^[8]^ Patients with CKD stage 3b and above have higher bone marrow fat content, reduced BMD, and increased risk of vertebral fractures, which may be related to the involvement of sclerostin.^[9]^

The 2009 Kidney Disease: Improving Global Outcomes (KDIGO) guidelines indicate that patients with CKD stages 3 to 5 and concomitant CKD-MBD with reduced BMD and fragility fractures should be designated as having CKD-MBD with low BMD. Patients with CKD have a high prevalence of OP, an increased incidence of bone fractures, especially hip fractures, and ultimately increased morbidity and mortality.^[10]^ Enhanced monitoring is particularly important to prevent and control falls and fractures in patients with multiple risk factors.^[11]^ CKD-MBD, different from osteoporosis in pathophysiology, is associated with altered mineral metabolism and an imbalance between pro-and anti-calcification factors that lead to high or low turnover bone disease in CKD patients.^[12]^

Nutritional vitamin D (NVD) is an micronutrient in the human body and a major fat-soluble vitamin that plays an essential role in human health. NVD helps the body absorb calcium and phosphorus, maintains bone health, and supports normal immune and nervous system function.^[13,14]^ Vitamin D deficiency is highly prevalent in the general population, with 37% of the global population having 25-hydroxyvitamin D (25(OH)D) levels under 20 ng/mL, and even up to 6.7% of individuals having 25(OH)D levels below 10 ng/mL, while the mean level of 25(OH)D did not reach 30 ng/mL, making this a major global health problem.^[15]^The vitamin D workshop consensus on vitamin D nutrition guidelines estimated that over 50% of older and younger adults have vitamin D deficiency or insufficiency. Even in countries with adequate sun exposure, over 1 billion people are considered to have vitamin D insufficiency or deficiency.^[16,17]^ In some cohort studies of CKD, up to 80% of patients had vitamin D deficiency despite differences in the definitional criteria used and the clinical characteristics of the study population.^[18,19]^ A very important physiological role of NVD is to maintain mineral-bone metabolic homeostasis by regulating calcium homeostasis. Vitamin D deficiency is also associated with the development or exacerbation of disorders outside the bone, such as skin, respiratory, endocrine, renal, cardiovascular, psychiatric, and neurodegenerative disorders.^[15,20]^ 25(OH)D regulates the activity of the innate and adaptive immune responses by acting on vitamin D receptors.^[21]^ Associations have been observed between low serum 25(OH)D levels and an increased risk of several immune-related diseases and disorders, including psoriasis, type 1 diabetes, multiple sclerosis, rheumatoid arthritis, tuberculosis, sepsis, respiratory infections, and COVID-19.^[21]^ Moreover, vitamin D affects the lungs and immune system during the fetal and neonatal periods, and 25(OH)D deficiency during pregnancy is associated with an increased incidence of postnatal asthma.^[22]^

## 2. Vitamin D physiological metabolism

The term vitamin D is used to describe a variety of vitamin D compounds and can lead to misunderstandings in the literature and in clinical practice. When discussing treatment and supplementation, references to vitamin D refer only to vitamin D_2_ or vitamin D_3_.^[4,19]^ Vitamin D_2_, also known as ergocalciferol, is formed in yeast and plants after UV exposure.^[23,24]^ Vitamin D_3_, also known as cholecalciferol,^[25]^ is the principal form of vitamin D.^[15]^ In addition to vitamin D from food sources, 7-dehydrocholesterol in the epidermis is converted to previtamin D3 which is thermally isomerize to vitamin D_3_,^[26]^ when exposured to sunlight or ultraviolet light at a wavelength of 290 to 320 nm.^[14,18,25,27,28]^ Vitamin D_3_ is mostly found in marine fish, animal liver, egg yolk, lean meat, skim milk, cod liver oil, cheese, nuts, and seafood.^[25,27]^ Both types of vitamin D have the same physiological role and are collectively referred to as NVD.^[29]^

Two chemical transformations are required for vitamin D to become fully bioactive. Ergocalciferol and cholecalciferol, obtained from both the dietary and dermal routes, are bound to the carrier vitamin D binding protein (DBP) in the blood and are transported to the liver. They are first transformed into 25(OH)D_2_ and 25(OH)D_3_, the major storage forms of vitamin D, by 25-hydroxylase in the endoplasmic reticulum and mitochondria of hepatocytes. 25(OH)D_2_ and 25(OH)D_3_ are again bound to DBP before being transported to the kidney and filtered through the glomeruli.^[28]^ 25(OH)D then travels to the proximal tubules of the kidney, where it is enzymatically metabolized to 1,25-dihydroxyvitamin D (1,25(OH)_2_D), the bioactive form of vitamin D. 1,25(OH)_2_D_3_ is transported by DBP to target organs, such as the small intestine and bone, where it binds to the vitamin D receptor (VDR) and enters the nucleus to exert its corresponding biological effects.^[30]^ In contrast to hepatic hydroxylation, renal hydroxylation is regulated by several factors, including calcium, phosphate, PTH, and fibroblast growth factor (FGF) 23, which is produced by osteocytes and osteoblasts.^[31]^ NVD has a long half-life of about 2 to 3 weeks; in the event of a toxic reaction, treatment requires several weeks.^[17,29]^ Vitamin D metabolites are inactivated in the kidney by 24-hydroxylase, a process regulated by FGF23 and calcitriol.^[32]^ Vitamin D is primarily stored in adipose tissue; lean individuals have lower storage capacity and are susceptible to vitamin D deficiency, whereas obese patients require higher doses of vitamin D to meet their physiological needs.^[33]^

## 3. Unique aspects of vitamin D in kidney metabolism

In CKD, 1,25(OH)_2_D deficiency is inevitable and leads to hypocalcemia and secondary hyperparathyroidism (SHPT), which are the primary causes of secondary OP.^[17]^ After renal failure, intrarenal 1α-hydroxylase activity decreases with renal tissue loss.^[31]^ 24,25(OH)D levels are even lower in dialysis patients than in the normal population. However, many extrarenal tissues also synthesize 1α-hydroxylase and produce 1,25(OH)_2_D locally, including the pancreatic islets, prostate, colon, breast, macrophages, malignant cells, immune cells, and vascular smooth muscle cells.^[29]^ The likely mechanism for extrarenal synthesis of 1,25(OH)_2_D remains intact in CKD patients. Moreover, when 1,25(OH)_2_D is reduced, the role of this mechanism increases in a compensatory manner.^[19]^ Extrarenal 1α-hydroxylase activity is dependent on the amount of its substrate 25(OH)D, and the hormones that regulate intrarenal 1,25(OH)_2_D have no effect on extrarenal 1α-hydroxylase.^[29]^ In addition, increased levels of PTH promote 24,25(OH)D enzyme activity, increase 25(OH)D degradation, and exacerbate 25(OH)D deficiency.^[29]^

In patients with CKD, a feedback loop exists between FGF23 and vitamin D. Osteocytes sense elevated blood phosphorus and compensate by increasing the secretion of FGF23. Elevated FGF23 further inhibits renal 1α-hydroxylase activity and expression, and 24-hydroxylase expression is also induced, resulting in reduced 1,25(OH)_2_D degradation.^[31,34]^ Dietary phosphate intake decreases renal 1α-hydroxylase activity and its mRNA expression. Prolonged phosphate retention and increased FGF23 may be the primary mechanisms inhibiting 1α-hydroxylase activation.^[17,29]^ In addition to these factors, 1α-hydroxylase activity may also be partially inhibited by PTH or uremic toxins.^[29]^ In the kidney, the interaction between FGF23 and the FGFR1/klotho complex results in decreased phosphate reabsorption by internalization and degradation of the sodium phosphate-phosphate cotransporter. This suppresses vitamin D formation by reducing 1α-hydroxylase expression and increasing calcium reabsorption in the distal tubule by upregulating calcium-selective channel proteins.^[10]^

The blood level of full-length or PTH usually remains normal until the estimated glomerular filtration rate falls to approximately 45 mL·min^-1^·1.73 m^-2^, then begins to increase.^[10]^ 25(OH)D levels begin to fall later than intact PTH levels after eGFR falls below 40 mL·min^-1^·1.73 m^-2^.^[34]^ Serum calcium levels do not decrease until GFR falls below 20 mL·min^-1^·1.73 m^-2^, while serum phosphate usually remains normal until GFR is 20 mL·min^-1^·1.73 m^-2[10]^ (Table 1).

**Table 1:** eGFR values at which renal disease indicators become abnormal.

Measurement indicator	Borderline eGFR for the indicator to be abnormal
Full-length parathyroid hormone	45 mL·min^-1^·1.73 m^-2^
25(OH)D	40 mL·min^-1^·1.73 m^-2^
Serum calcium	20 mL·min^-1^·1.73 m^-2^
Serum phosphate	20 mL·min^-1^·1.73 m^-2^

eGFR = estimated glomerular filtration rate.

## 4. Definition of vitamin D deficiency and risk factors

The definition of vitamin D deficiency has evolved over the past decade. Most clinicians cite the recommendations of the International Osteoporosis Foundation guidelines: a 25(OH)D concentration of <20 ng/mL is defined as deficiency, a 25(OH)D concentration of 21 to 29 ng/mL as insufficiency, and a 25(OH)D concentration of >30 ng/mL as normal.^[25,35,36]^ It is unclear whether the definition of vitamin D deficiency should differ in the context of CKD. Vitamin D deficiency is more severe in patients on maintenance hemodialysis, where levels can be lower than 5 ng/mL.^[33]^ It is hoped that more targeted and interventional studies in the future may contribute knowledge to reveal “personalized” target 25(OH)D levels. Serum 25(OH)D is considered the best indicator of vitamin D status because it has a longer biological half-life than 1,25(OH)_2_D and circulates at higher concentrations.^[18,28]^ The Kidney Disease Outcomes Quality Initiative guidelines recommend using a 25(OH)D level of 30 ng/mL as the normal reference value for non-dialysis CKD patients, which can also be considered a suitable therapeutic target for patients on dialysis. Furthermore, sunlight exposure varies seasonally, and vitamin D levels also exhibit seasonal variations; thus, 25(OH)D should be measured twice per year, once in late summer and once in late winter. If persistent hypercalcemia or hyperphosphatemia is discovered, monitoring frequency should increase.^[37]^ Different types of assays are available for measuring 25(OH)D. High-performance liquid chromatography is not widely used because it is expensive, slow, and requires specialized knowledge and instrumentation. The most common method in most studies is the DiaSorin Molecular automated chemiluminescence assay, which is co-specific for 25(OH)D_2_ and 25(OH)D_3_.^[18]^ 25(OH)D levels are expressed as ng/mL or nmol/L, and a simple formula can be used to convert between these units: 1 nmol/L = 2.5 ng/mL.^[38]^

Common causes of vitamin D deficiency include inadequate sun exposure, unbalanced diet, and some diseases and medications. The causes of 25(OH)D_3_ deficiency in patients with CKD and dialysis patients are more complex and include advanced age, female gender, obesity, proteinuria, insufficient physical activity, diabetes, reduced vitamin D skin synthesis, vitamin D receptors, and the use of calcineurin inhibitors.^[17]^

## 5. Types of vitamin D^[39,40]^

Generally, vitamin D is divided into 3 groups (Table 2). The first group is composed of NVD, namely ergocalciferol and cholecalciferol. The second group is composed of bioactive vitamin D analogs in the form of nonselective vitamin D receptor agonists such as calcitriol (first-generation) and alfacalcidol (second-generation). The third group is composed of selective vitamin D receptor agonists, such as paricalcitol (third-generation), maxacalcitol (second-generation), and doxercalciferol. Selectivity refers to highly specific action on the parathyroid glands but not on the intestine or bones, allowing reduction of serum calcium and phosphorus blood concentrations.^[33]^

**Table 2:** Types of vitamin D.

Group	Vitamin D_2_	Vitamin D_3_
Novel vitamin D receptor agonists	Paricalcitol (19-nor-1,25-dihydroxyVD)	Maxacalcitol (22-oxa-1α,25dihydroxyVD3)
	Doxercalciferol (1α-hydroxyergocalciferol)	Falecalcitriol (F6-1α,25dihydroxyVD3)
Bioactive vitamin D	–	Calcitriol
		Alfacalcidol
Nutritional vitamin D	Ergocalciferol	Cholecalciferol

VD = vitamin D.

Extended-release calcifediol (ERC), an orally administered prohormone of calcitriol, has become an innovative treatment option for CKD3-4 patients who develop progressive SHPT.^[41]^ ERC raises serum 25(OH)D levels gradually, with no increase in serum phosphorus, calcium and FGF 23.^[42]^ Meanwhile, ERC was able to reduce PTH levels effectively without oversuppression.^[43]^

There were several differences between ERC and NVD: (a) ERC is more bioavailable and water soluble; (b) ERC does not need to be metabolized by the liver; (c) ERC is closely bound with DBP in serum to improve the pharmacological effect of serum 25(OH)D.^[44]^

## 6. A shift in the understanding of vitamin D

Here, we review our historical understanding of vitamin D deficiency in CKD. Over time, there has been considerable evolution on when treatment should be initiated, how vitamin D should be supplemented, which form of vitamin D to supplement, what dosage should be used, and which route of treatment should be adopted for non-dialysis CKD patients and patients on dialysis; but, no consensus position has been established.

At early stages, CKD results in defective vitamin D activation in the kidney, resulting in hypocalcemia and hyperphosphatemia and a compensatory increase in parathyroid cells and PTH secretion, leading to SHPT. Serum calcium concentration is the main determinant of PTH release. The parathyroid glands have calcium-sensing receptors (CaSR) that sense changes in plasma calcium concentration and regulate PTH secretion. Persistent low calcium leads to parathyroid cell hyperplasia and downregulation of parathyroid VDRs and CaSR. SHPT caused by increased PTH secretion causes vascular calcification of coronary vessels and other peripheral vessels, causing ischemic cardiovascular events and heart failure.^[45]^ If CaSR expression is reduced by overgrowth of the parathyroids, even hypercalcemia cannot suppress PTH secretion. Eventually, elevated PTH increases the risks of osteoporotic fracture and shrinking man syndrome.^[10]^ Given the side effects of excessive PTH, the primary reason for vitamin D treatment of patients with kidney disease is to reduce PTH levels.^[46]^

In 2009, the KDIGO initiative published the first set of guidelines for the diagnosis and treatment of patients with CKD-MBD, highlighting its importance. The incidence of hypercalcemia, hyperphosphatemia, vascular calcification, and adynamic bone disease has also increased significantly due to the aggressive clinical use of bioactive vitamin D and vitamin D receptor agonists and excessive suppression of PTH.^[17]^ Calcium balance is whole-body calcium retention or deficit calculated by subtracting total body calcium losses from total calcium inputs. A positive balance may increase vascular calcification and cardiovascular events, while a negative balance may increase the risks of osteoporosis and fracture.^[34]^

Nephrologists became aware of the need for measured use of calcitriol, explicitly avoiding premature and excessive treatment with bioactive vitamin D (including VDR receptor agonists). The observation that overt or even latent hypercalcemia was noxious in patients with CKD or on dialysis led to large-scale research into calcium-free phosphate binders and alternative drugs that effectively controlled parathyroid hyperplasia without increasing the propensity for hypercalcemia. However, even the novel vitamin D analogs are not neutral with regards to the risk of hypercalcemia development.^[47]^ Cinacalcet, a variant G protein-coupled receptor modulator that activates CaSR, efficiently decreases serum calcium and PTH in CKD-associated SHPT. Long-term use of cinacalcet reduces and improves the high bone turnover rate in regular hemodialysis patients with SHPT.^[45]^

The 2017 KDIGO CKD-MBD guidelines recommend against calcitriol and vitamin D analog supplementation in patients with CKD without dialysis because over supplementation can lead to hypercalcemia and hyperphosphatemia, which may promote vascular calcification.^[48]^ For treatment of severe and progressive hyperparathyroidism, calcitriol or bioactive vitamin D analogs should be started at a low dose and then titrated based on the response of PTH.^[48]^ The different effects of calcitriol and paricalcitol on vascular calcification are associated with the different regulatory effects on the Wnt/β-catenin pathway. Calcitriol activates the Wnt/β-catenin pathway, upregulates the expression of osteogenic markers such as BMP2, Ruru2, Msx2, and OC, and increases calcification, whereas paricalcitol downregulates Wnt/β-catenin signaling and osteogenic marker expression and decreases calcification.^[49]^ This comparison between calcitriol and paricalcirol is only based in in vitro results, and never confirmed in any clinical data.

For many years, the treatment philosophy for patients with advanced CKD has been based on what may be 2 misconceptions: (1) bioactive vitamin D alone can completely correct vitamin D deficiency, and (2) bioactive vitamin D therapy should be given only in the presence of SHPT or osteochondrosis.^[38]^ Both of these concepts are now being challenged.

NVD supplementation has 2 advantages. First, NVD is unlikely to cause hypercalcemia unless high doses are consistently given because its 1𝛼-hydroxylase-mediated activation process is regulated by many factors, such as PTH, FGF23, and 24-hydroxylase. Second, after conversion to 25(OH)D, NVD forms a complex with DBP, which has a long half-life.^[50]^ Furthermore, long-term cholecalciferol supplementation in hemodialysis patients can reduce the dose of erythroid-stimulating agent, and decrease brain natriuretic peptide plasma levels and left ventricular mass index, an independent benefit from antihypertensive therapy such as angiotensin-converting enzyme inhibitors, angiotensin II receptor blockers, and statins.^[51]^ The Endocrine Society Clinical Practice Guideline recommend prescribing vitamin D supplements to prevent falls, but no excess of the recommended daily to avoid cardiovascular disease and death or to improve quality of life.^[52]^

## 7. Supplementation with NVD

According to clinical guideline recommendations for vitamin D administration, patients with vitamin D deficiency requiring supplementation are to be given an exact and controlled amount of IU, and vitamin D3 appears to be the most reasonable option, probably because of its greater lipophilicity and slower pharmacokinetic elimination.^[15]^ Compared to intramuscular administration, oral administration can increase patient blood 25(OH)D levels 3-fold after 1 month.^[20]^ The response to vitamin D_3_ supplementation differs among individuals, who can be classified as high, medium, or low responders. Vitamin D low responders are the most sensitive to vitamin D deficiency and require higher daily doses of vitamin D_3_ supplementation (approximately 50–100 µg) to maintain optimal endocrine vitamin D activity.^[22]^ VDR gene polymorphisms can affect individual susceptibility and response to vitamin D supplementation of patients with osteoporosis. 25(OH)D has significant dose-dependent effects on the rs1544410, rs731236, and rs11568820 genotypes, especially the rs731236 point mutation genotype A/A of the vitamin D receptor gene, which is strongly associated with 25(OH)D deficiency.^[20]^ Most studies of vitamin D supplementation use doses of 400 to 1000 IU/day, and low doses of vitamin D are safe, but >4000 IU/day leads to more falls and fractures.^[14]^ Every 100 IU/ D of vitamin D intake increases serum 25(OH)D by approximately <1 ng/mL.^[52]^

A 2021 randomized controlled trial of 60 maintenance hemodialysis patients found that the treatment group received 200 IU oral vitamin D_3_ per month and had significantly higher blood 25(OH)D and fetal globulin-A levels after 3 months.^[53]^ A study used receiver operating characteristic curve analysis to derive optimal cutoff serum 1,25(OH)_2_D values of ≤12.5 pg/dL (sensitivity 80.8%, specificity 70.0%) and ≤11.9 pg/dL (sensitivity 71.6%, specificity 70.8%) for predicting aortic calcification and mitral valve calcification, respectively. 1,25(OH)_2_D deficiency was independently associated with aortic calcification and mitral valve calcification, suggesting that serum 1,25(OH)_2_D levels may be a potential biomarker for aortic calcification and mitral valve calcification in these patients.^[54]^ Vitamin D_3_ supplementation has been shown to treat vitamin D deficiency in dialysis patients and to elevate fetuin-A levels,^[53]^ and reduce pulse wave velocity.^[55]^ Multivariate logistic regression analysis concluded that 25(OH)D levels (odds ratio: 0.895, 95% confidence interval: 0.828–0.968, *P* = .005) and age (odds ratio: 1.140, 95% confidence interval: 1.088–1.194, *P* < .001) were independent risk factors predictive of the development of peripheral atherosclerosis, with lower serum 25(OH)D levels and greater age contributing to atherosclerosis.^[56]^

In a large meta-analysis of 22 studies, including 17 observational studies and 5 randomized controlled trials, the effect of NVD supplementation on CKD patients, dialysis patients, and renal transplant recipients was investigated. The results showed that NVD was associated with a significant increase in 25(OH)D levels in CKD and dialysis patients, while no correlation was found in renal transplant recipients.^[28,57]^ Moreover, vitamin D is more effective when given intravenously before weaning from dialysis than when taken orally at home.^[34]^ Nevertheless, in non-dialysis CKD patients, we still have insufficient evidence correlating that vitamin D2 and vitamin D3 are equally effective in maintaining serum 25(OH)D levels.^[52]^ Some of studies have shown that cholecalciferol may be superior to ergocalciferol in increasing serum 25(OH)D levels and is recommended as the first choice for supplementation.^[58]^ Furthermore, ergocalciferol is less effective in increasing 25(OH)D plasma concentrations due to its plant origin, shorter half-life, a methyl group on C24 that reduces conversion to 25(OH)D_2_, and lower affinity for binding proteins.^[18]^ In addition, the toxicity threshold of vitamin D is poorly defined for maintenance hemodialysis patients. Although the toxicity threshold of vitamin D is 150 ng/mL or 100ng/mL in the general population,^[52]^ it seems advisable by some researchers not to exceed the threshold of 80 ng/mL in maintenance hemodialysis patients.^[33]^ More research is needed to find out.

## Author contributions

**Investigation:** Yingjing Shen.

**Methodology:** Yingjing Shen.

**Resources:** Yingjing Shen.

**Supervision:** Yingjing Shen.

**Writing – original draft:** Yingjing Shen.
